# Influencing Factors on the Outcome and Prognosis of Patients With HBV Infction: Seven Years Follow-up

**DOI:** 10.5812/hepatmon.8743

**Published:** 2013-07-08

**Authors:** Shahnaz Sali, Seyed Moayed Alavian, Graham R Foster, Hossein Keyvani, Leila Mehrnoosh, Navid Mohammadi

**Affiliations:** 1 Infectious Diseases and Tropical Medicine Research Center, Shahid Beheshti University of Medical Sciences, Tehran, IR Iran; 2 Baqiyatallah Research Center for Gastroenterology and Liver Disease, Tehran, IR Iran; 3 Queen Mary, University of London, the Liver Unit, London, UK; 4 Department of Virology, Tehran University of Medical Science, Tehran, IR Iran; 5 Department of Community Medicine, Faculty of medicine, Tehran University of Medical Sciences, Tehran, IR Iran

**Keywords:** Hepatitis B, Hepatitis B Surface Antigens, Longitudinal Studies, Confounding Factors (Epidemiology)

## Abstract

**Background:**

Hepatitis B virus (HBV) is one of the most common chronic viral infections in the world. Iran has a low to intermediate HBV prevalence and approximately 1.5 million people are living with HBV infection. The impact of HBV in Iran is unknown and given the very low levels of alcohol consumption, this region provides an opportunity to examine the impact of isolated chronic HBV infection.

**Objectives:**

To examine and evaluate outcome and prognosis of HBV in Iran.

**Patients and Methods:**

A longitudinal cohort study dating from 2003-2010 was performed. The patients were assessed six months after their first visit and then during periodic visits for the subsequent seven years. The patients’ medical history, route of diagnosis of infection, family history, and liver diseases status including: carrier state of HBV, chronic HBV, cirrhosis, and HCC were recorded. Descriptive and analytic statistics were performed, using SPSS software version 18.

**Results:**

275 HBsAg positive patients, who had completed a 7 year follow up period, were selected. The annual incidence rate for chronic hepatitis B in inactive carrier states and cirrhosis were 0.46% and 0.2% respectively. Over seven years, the rate of inactive carriers decreased by eight percent (They turned into chronic HBV or became HBSAg negative). No significant association was found between HBSAg seroclearance, HBeAg seroconversion and the outcome in the end of each year of follow up. Different treatment regimens did not have any statistically significant difference regarding HBeAg seroconversion. There was no significant association between the outcome and different habitual characteristics, especially smoking, as well as family history on HBsAg, HBsAb, HBeAg, and Anti-HBeAg. Values of platelets and ALT showed a significant change during the follow ups. Annual incidence rate of HCC in the present study was in the range of other studies.

**Conclusions:**

These data confirm and extend data from other populations showing a low incidence of significant change in chronic HBV infection in short term with good responses to currently available therapeutics.

## 1. Background

Hepatitis B virus (HBV) is one of the most common chronic viral infections in the world (1). In the Middle East, HBV infection is of an intermediate endemicity with chronic HBV carriage rate of 2-5% among the general population (2). Worldwide more than 400 million individuals are chronically infected with HBV, and they are at the risk of developing hepatocellular carcinoma (HCC) and cirrhosis (3). However, the incidence rates of developing HCC and cirrhosis vary widely around the world. Several factors have been identified that could influence the rate of these complications resulting from HBV infection. These include sex with higher rates of HCC found in men than women, geographic areas with higher rates found in Eastern and South East Asia and sub-Saharan Africa (3), and environmental factors such as the presence of aflatoxin on food, ethnicity, heavy alcohol consumption, as well as smoking (4). Despite the availability of effective vaccines and effective antiviral medications, approximately one million people die annually of acute and chronic HBV infections (5, 6). Several viral factors appear to strongly influence the outcome in HBV infection, including HBV genotype, DNA levels over time and specific HBV viral mutations (7). Mutations in the HBV surface (S), precore (PC) and basal core promoter (BCP) genes are observed frequently in patients with HBV infection , and studies show that these mutations are associated with the clinical outcomes of HBV disease. There are 8 HBV genotypes in the world and incidence rate of HCC and cirrhosis in every genotype are varied (7-9). Genotype D is the most common genotype in Iran and according to WHO classification, Iran has a low to intermediate HBV prevalence, and it is estimated that about 1.5 million people are living with HBV infection (10).

## 2. Objectives

In Tehran Province HBV infection is estimated to affect 2-3% of the population (10). But factors influencing outcome have not been described. Here we describe, for the first time the outcome of a cohort of patients followed up for seven years.

## 3. Patients and Methods

### 3.1. Type of Study and Research Environment

This longitudinal study was performed from 2003 to 2010 on a group of 275 patients aged between 9-84 years old who were diagnosed with HBs Ag over a period longer than six months. The cases were selected from a total of 800 patients with positive results for HBs Ag referred to the Tehran Hepatitis Centre (THC: the outpatient clinic of the Baqiyatallah Research Center for Gastroenterology and Liver Diseases) and had completed seven years of follow up. They had been admitted due to incidental detection of HBs Ag during blood donation, routine check-ups, and work-ups for nonliver diseases or referred from other outpatient clinics. Immunosuppressed patients (who had undergone dialysis or received immunosuppressive drugs), those with concurrent hepatitis C virus, HIV infection or other liver diseases (Wilson disease, autoimmune hepatitis, α-1-antitrypsin deficiency) and those not willing to participate in the survey (n = 158) were excluded from the study. Informed consent was obtained from all patients. Medications being used in the process of treatment were conventional Interferon (IFN) and Direct Antiviral (DAV) therapies, including Lamivudine, Adefovir or combination of Lamivudine and Adefovir.

### 3.2. Follow up

The patients were assessed six months after their first visit and then during periodic visits for the subsequent seven years. The patients’ medical history, route of diagnosis of HBV infection, and family history of liver diseases including: carrier state of HBV, chronic HBV, cirrhosis, and HCC were recorded. During each visit, Serum aspartate aminotransferase (AST) and alanine aminotransferase (ALT) levels, Blood biochemical tests [including fasting blood glucose (FBS), triglyceride (TG) and cholesterol (Chl), Complete blood count (CBC), Serological HBV markers, and physical examinations were performed. Serum aspartate aminotransferase (AST) and alanine aminotransferase (ALT) levels were evaluated using standard methods (upper limit of normal, 40 IU/L). The viral markers (HBs Ag, anti-HBs, HBeAg, and anti-HBe) were measured using the ELISA method (11). Serum HBV DNA level was quantitatively assessed in Keyvan Viral Special laboratory by real Cobas Amplicor and Cobas TaqMan from Roche Diagnostics. The conversion factor for Cobas Amplicor is 5.26 and for Cobas TaqMan is 5.82. The lower detection limit (Cut off) for Amplicor is 200 copies /mL (38 IU/mL) and 30 copies (6 IU/mL) for TaqMan. Patients were selected for treatment according to standard criteria: serum aminotransferase level of at least 1.5 times of normal upper limit; HBV Viral load more than 10,000 copy/ml (2000 IU/ml), and evidences of necrotic-inflammatory activity in the liver biopsy samples. All treated patients (240) and some of untreated (10 patients) underwent liver biopsy. Histological assessment revealed mild and moderate hepatitis in the treated patients, and minimal hepatitis in the untreated ones. Terminology, definitions and the diagnosis criteria were established in accordance with the EASL guidelines 2008 (12-16):

- Inactive carrier state was defined as having a detectable HBs Ag, an undetectable HBeAg, a serum HBV DNA level < 2000 IU/ml, and persistently normal ALT levels more than 6 months.

- Patients with chronic HBV, were defined as having detectable HBsAg, either HBeAg positive or negative, a serum HBVDNA level >2000 IU/ml, and elevated transaminase level.

- HBSAg sero-clearance was defined as two consecutive negative serums HBsAg at least 6 months apart, which were maintained to the end of the study, normal ALT level, and undetectable serum HBVDNA level.

- HBeAg sero-conversion was defined as two consecutive negative HBeAg and positive HBeAb.

- Liver cirrhosis was based on the ultrasonographic findings supported by liver histology, thrombocytopenia, or endoscopic findings of esophageal varices.

- Hepatocellular carcinoma (HCC) was diagnosed by pathologic findings.

### 3.3. Data Analysis

The mean ± standard deviation (SD) was used for the description of quantitative variables. The Student t-test and one-way ANOVA were used for comparing quantitative variables, and the chi-square test was used for comparisons involving categorical variables. Repeated measures analyses were performed for changes during seven years follow up in different numerical variables. Logistic regression analyses were performed for assessment of effective factors on the outcome. Differences or correlations (Pearson’s or Spearman’s) with P < 0.05 were considered statistically significant. Descriptive and analytic statistics were performed using SPSS software version 18 (IBM Corporation, NY, The U.S.A). The study protocol conforms to the ethical guidelines of the declaration of Helsinki.

## 4. Results

### 4.1. Demographics

The 275 cases were selected from a total of 800 patients with positive results for HBs Ag who was referred to the Tehran Hepatitis Centre (THC) as they had completed seven years of follow up. Their details are outlined in Table 1. Patients with different types of clinical situation at first visit, had different conditions of age (P < 0.001), WBC (P = 0.004), platelets (P < 0.001), PT (P < 0.001), AST (P = 0.024), ALT (P = 0.013), protein (P = 0.019), viral load (P < 0.001). After seven years follow up, platelets count showed a significant difference between different groups of outcome, while no other significant differences were noted. The family history of patients showed that 55% had a positive history of hepatitis B for at least one of their first degree relative which indicates the importance of familial transmission of HBV, in Iran. In general, for each respective clinical situation, there was no statistically significant association between the outcome and different family history characteristics. Smoking, alcohol consumption, and opium intake were assessed, and 72% of patients (198) denied such habits. Others had a history of consumption of at least one substance: 51 (18.5%) smoked, 15 (5.45) drank, and 11 (4%) were addicted to opium. There was no association between disease outcome and addictive history.

### 4.2. Clinical Outcome

Data on outcome are summarized in Table 2, as follows. The outcome of patients were assessed on 6 categories; inactive carrier state (group A), chronic hepatitis (group B), cirrhosis (group C), HCC, death, and finally negative cases. At first visit, there were 51 (18.5%) inactive carriers, 199 (72.4%) chronic hepatitis, and 25 (9.1%) cirrhosis. After seven years 2 (2.9%) ‘inactive carriers’ became HBS Ag negative, and 4 (2.01%) cases with active disease lost HBS Ag, 20 (39%) cases developed active disease, and 6 (3.05%) cases with active disease developed cirrhosis, and 20 (39%) cases moved from Group A to Group B. Under such circumstances, the total number of cases in Group B increased to 208 cases. Two patients with cirrhosis died. Table 3 indicates Mean, SD, and 95% confidence interval of survival time for each status change. Incidence of each outcome was calculated which shows that annual incidence rate was 0.46% for chronic hepatitis, 0.31% for negative HBsAg; 0.2% for cirrhosis, 0.1% for HCC, and 0.1% for death. Over seven years, the rate of inactive carriers decreased by 8% (most of them turned into chronic hepatitis), while increasing rate of negative HBsAg was 2.2%. There was a significant correlation between the outcome of patients at the first visit and 7th year (Spearman’s rho = 0.725, P < 0.001). To evaluate survival of HBV infection, Kaplan-Meier survival, and COX Regression graph were used. Different events were considered as follows;

A: Inactive carrier to chronic hepatitis, cirrhosis, HCC, HBS Ag negative, and death.

B: Chronic hepatitis to cirrhosis, HCC, death, and HBS Ag negative.

C: Cirrhosis to HCC and death.

D: HBeAg sero-conversion.

E: HBsAg sero-conversion.

**Figure 1.**
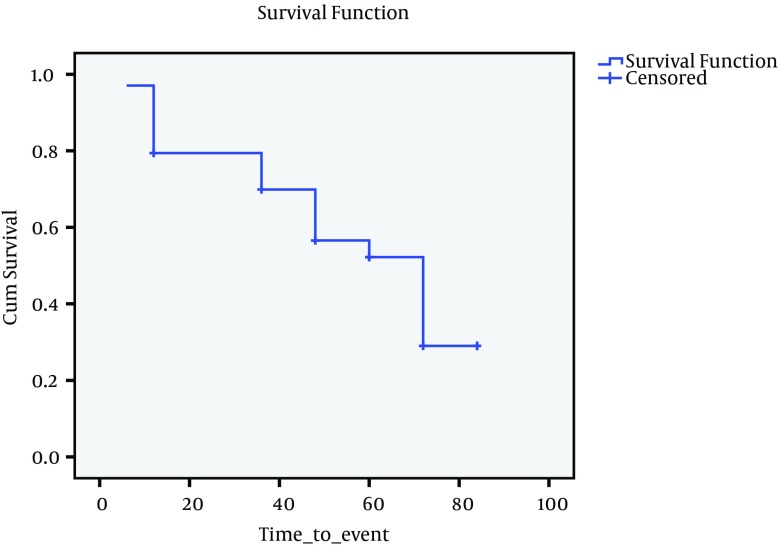
Kaplan-Meier Graph for Chronic to Cirrhosis

**Figure 2.**
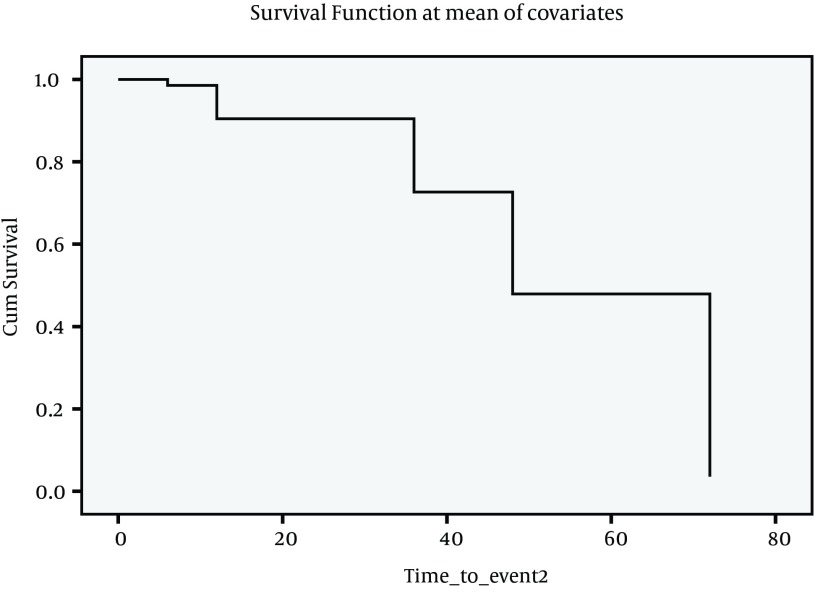
COX- Regression Analysis Graph for Inactive Carrier State to Chronic HBV

**Figure 3.**
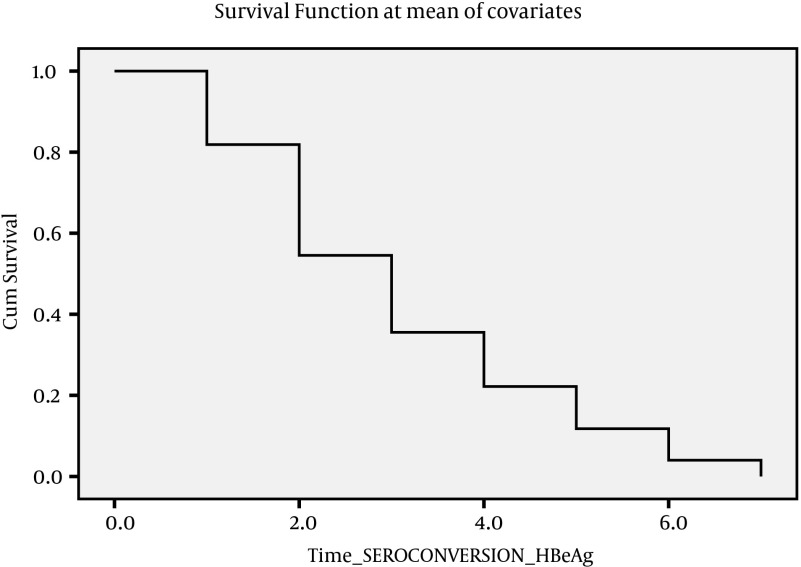
COX- Regression Analysis Graph for HBe Sero-Conversion

**Figure 4.**
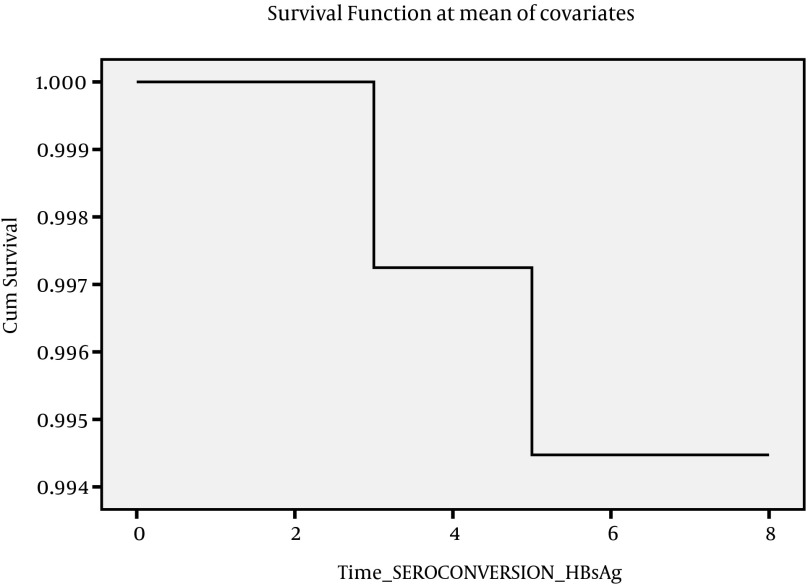
COX - Regression Analysis Graph for HBsAg Sero-Conversion

Since there were insufficient cases for events described above, it seemed more feasible to consider four events as indicated in Figures 1, 2, 3 and 4. Figure 1 presents Kaplan-Meier for an event; chronic hepatitis B to cirrhosis, and figures 2 to 4 COX regression analysis for three events; Inactive carrier state to chronic hepatitis B, HBeAg sero-coversion and HBsAg sero-coversion (sero-clearance). 

### 4.3. Effect of Treatment Regimens

Different medication regimens and treatments were used as shown in Table 4. Treatment regimens were assessed in two groups; one containing Interferon and another Direct Antiviral (DAV) drugs including Lamivudine, Adefovir or combination of Lamivudine and Adefovir. Association between different therapeutic regimens and the outcome was assessed, as well. Some patients may have received several rounds of treatment during 7 years follow up, and that is why a total of 489 treatments are presented for a total of 275 patients. Of 79 cases receiving Interferon, only 1 case HBSAg seroconverted to HBSAb (1.2%). No cancers developed in this group and only 2 cases converted to cirrhosis (2.5%). Of 279 cases receiving Lamivudine, 4 and of 21 receiving Lamivudine + Adefovir, 2 cases became HBSAg seroclearance (total 6 cases, 2.16%, CI 95%: 1.2 – 3.12%) and “HBeAg (+) to HBeAb (+)” seroconversion occurred in 17 cases (21.8%, CI 95%: 16.7-26.9%) that received Interferon (6 cases), Lamivudine (6 cases) and Lamivudine + Adefovir (5 cases).

### 4.4. HBV Markers:

Assessment of HBsAg showed that 267 of 275 cases still had positive findings for HBsAg after seven years follow up (97.1%)(CI 95%: 94.3-98.7%). At first visit, HBeAg had positive results in 78 patients (28.4%) (CI 95%: 23.1-34.1%), and after seven years follow up, 42 cases still showed positive findings for HBeAg (15.2%) (CI 95%: 12.51-18.12%). In a logistic regression analysis (and after adjustment for sex, age, weight and height) there was no effect of family history, and no personal habits on HBsAg, HBsAb, HBeAg, and Anti-HBeAg. Seroconversion from HBsAg (+) to HBsAb (+) (HBSAg seroclearance) and HBeAg (+) to HBeAb (+) (HBeAg seroconversion) happened in 6 (2.16%, CI 95%: 1.2 – 3.12%) and 17 (21.8%, CI 95%: 16.7-26.9%) cases, respectively.

There were 2 HCC cases and 2 mortality as the consequence liver diseases during seven years follow up. (Annual incidence rate was 0.1%). Repeated measures analysis was performed for assessment of changes in DNA load for HBV and different Para-clinical characteristics of patients during the follow up years. There was no significant change of DNA load of HBV, but values of platelets (P = 0.046) and ALT (P = 0.016) showed a significant change during the follow up.

**Table 1.** Patients with different types of clinical situation at first visit

	Sex (M/F)	Age (Mean ± SD)	Platelate^a^ (Mean ± SD)	PT (Mean ± SD)	AST (Mean ± SD)	ALT (Mean ± SD)	Viral load+ (Mean ± SD)
**Inactive Carrier**	37/14	39.6 ± 11.8	216.8 ± 56.7	12.9 ± 0.6	32.4 ± 18.1	42.8 ± 8.3	1,575 ± 1,360
**Chronic Hepatitis B**	142/57	41.4 ± 13.1	202.2 ± 54.6	13.3 ± 1.0	73.9±11.1	110.5 ± 72.5	148,041 ± 67,803
**Cirrhosis**	21/4	20.6 ± 13.2	112.2 ± 70.0	14.4 ± 2.1	76.8 ± 66.4	69.8 ± 58.4	6,193 ± 4,513
**Total**	200/75	42.8 ± 14.1	196.6 ± 62.7	13.3 ± 1.2	66.5 ± 28.1	94.3 ± 52.1	124,562 ± 25,650

^a^ X10^3 ^+, International Unit

**Table 2.** Frequency of Different Outcome During Follow up

	First visit	6 months	1^st^ year	2^nd^ year	3^rd^ year	4^th^ year	5^th^ year	6^th^ year	7^th^ year
**Inactive Carrier, No. (%)**	51 (18.5)	49 (17.8)	44 (16)	45 (16.4)	43 (15.6)	34 (12.4)	33 (12)	29 (10.5)	29 (10.5)
**CI 95%**	14.1 – 23.7	13.5 – 22.9	11.9 – 20.9	12.2 – 21.3	11.6 – 20.5	8.7 – 16.8	8.4 – 16.4	7.2 – 14.8	7.2 – 14.8
**Chronic Hepatitis, No. (%)**	199 (72.4)	201 (73.1)	204 (74.2)	203 (73.8)	204 (74.2)	210 (76.4)	209 (76.0)	211 (76.91)	208 (75.6)
**CI 95%**	66.7 – 77.6	67.4 – 78.2	68.6 – 79.3	68.2 –78.9	68.6 – 79.3	70.9 – 81.3	70.5 – 80.9	71.3 – 81.6	70.1 – 80.6
**Cirrhosis, No. (%)**	25 (9.1)	25 (9.1)	27 (9.8)	27 (9.8)	27 (9.8)	26 (9.8)	26 (9.5)	28 (10.4)	29 (10.5)
**CI 95%**	6.0 – 13.1	6.0 – 13.1	6.6 – 14.0	6.6 – 14.0	6.9 – 14.4	6.3 – 13.5	6.3 – 13.5	6.9 – 14.4	7.2 – 14.8
**HCC, No. (%)**	----------	----------	---------	---------	----------	1 (0.4)	----------	----------	1 (0.4)
**CI 95%**	--------	--------	--------	--------	--------	0.01 – 2.0	--------	--------	0.01 – 2.0
**Negative, No. (%)**	----------	----------	----------	----------	1 (0.4)	4 (1.5)	5 (1.8)	5 (1.8)	6 (2.2)
**CI 95%**	--------	--------	--------	--------	0.01 – 2.0	0.4 – 3.7	0.6 – 4.2	0.6 – 4.2	0.8 – 4.7
**Death, No. (%)**	----------	----------	---------	---------	----------	----------	--------	----------	2 (0.7)
**CI 95%**	--------	--------	--------	--------	--------	--------	---------	--------	0.09 – 2.6

**Table 3.** Survival Time for Each Status Change (Events in Month)

Event	Count	Mean	SD	Lower bound	Upper bound
**Inactive to negative**	2	83.73	0.194	83.35	84.11
**Inactive to chronic**	19	80.82	0.796	79.26	82.39
**Chronic to negative**	3	83.59	0.244	83.11	84.07
**Chronic to cirrhosis**	7	83.29	0.418	82.47	84.1
**Cirrhosis to death**	1	83.91	0.094	83.72	84.09
**Cirrhosis to HCC**	2	83.86	0.194	83.48	84.24
**HCC to death**	1	83.86	0.137	83.59	84.13

**Table 4.** Different Medications, Applied to Patients

Drugs (Treatment rounds )	1^st^ Drug	2^nd^ Drug	3^rd^ Drug	4^th^ Drug	5^th^ Drug	Total
**Interferon**	59	16	3	1	0	79
**Lamivudine**	162	78	28	10	1	279
**Adefovir**	7	54	35	10	4	110
**Adefovir+Lamivudine**	9	10	2	0	0	21
**Total**	237	158	68	21	6	489

## 5. Discussion

This was an observational cohort study performed on a cohort of 275 patients with HBV infection who were followed for a period of seven consecutive years. Most (200) were male which differs from previous Iranian studies (17). Among all cases 55% had a positive family history of hepatitis B indicating the importance of familial transmission of HBV as vertical transmission and horizontal in childhood in Iran (18). Smoking and alcohol consumption and opium were assessed and nearly 75% of cases had a negative history. As the information in this field was based on self-reporting and, taking into account the regulatory conditions in Iran for using alcohol and other illegal drugs, there is a possibility for underestimation in these areas. The low rates of HCC and conversion to cirrhosis may have been influenced by these low rates of alcohol and nicotine consumption. At the end of the follow up, HBsAg had still positive results in 267 patients while 2 cases died and 7 other patients converted to HBsAg negative (clearance rate = 2.01%). Clearance rate for HBeAg was calculated at around 21.8%. This rate is compatible with previous information from HBV infection in Iran (18). The most important difference between the present work and the previous studies in Iran lies in the fact that all patients in the current study received antiviral therapy. HBsAg seroclearance was found in 2.2% of treated patients and this differs from other studies. For example, in a study from France, 29% of cases that underwent interferon and had SVR developed HBsAg seroclearance (19). All of patients in this study had gone under conventional interferon and this might be a possible reason for the difference in the rates. On the other hand, in the present study, HBeAg seroconversion was seen in 21.8% of cases during seven years follow up. This rate is different from some other studies. In a study from China, the rate of HBeAg seroconversion was 40.2%, while mean time of follow up was less than 4 years (8). The rate of cirrhosis in the current study was annually 0.2%. Other studies on patients with Hepatitis B show different incidence rates. For example, three different studies from Taiwan have reported 0.9 (20, 21), and 0.07 (22), while other studies from Taiwan and Korea, have reported a rate of 1.6 (13, 23-25). Some European studies have reported different rates, as well: 0.01 (26), 3.8 (27, 28), and 9.7 (29, 30). These different rates can be derived from different types of patient and population in the studies. However, the annual incidence of HCC in the present study was less than 0.1%, which is in the range of other studies from different locations (from 0.02 (26, 31) to 3.7 (32)). Most patients received Lamivudine, while Adefovir and conventional interferon were used in lower priority by physicians. No significant association between treatment regimens and the outcomes could be seen. This may however be due to inadequate sample size/follow up period. Also we can clearly mention that our study is not a representative one on Iranian population but a sample of patients of a referral and tertiary center of hepatitis, so we do not claim that our results could be generalized to all Iranian population. This study indicates that chronic HBV outcome amongst Iranian patients with careful follow up is relatively benign. The annual incidence rate was 0.46% for chronic hepatitis, 0.31% for negative HBsAg; 0.2% for cirrhosis, 0.1% for HCC, and 0.1% for death. Over seven years, the rate of inactive carriers decreased by eight percent (most of them turned into chronic hepatitis) while increasing rate of negative HBsAg was 2.2%. There was a significant correlation between the outcome of patients at the 1st visit and 7th year (Spearman’s rho = 0.725, P < 0.001). It is possible that interventions with antiviral drugs improve chronic HBV outcome, and there may exist some kinds of diversity in viral genotype; a subject to be elaborated in future studies where sufficient data would be available to make definite conclusion.
